# Inflammatory Stimuli Responsive Non-Faradaic, Ultrasensitive Combinatorial Electrochemical Urine Biosensor

**DOI:** 10.3390/s22207757

**Published:** 2022-10-13

**Authors:** Antra Ganguly, Varun Gunda, Kevin Thai, Shalini Prasad

**Affiliations:** Department of Bioengineering, The University of Texas at Dallas, Richardson, TX 75080, USA

**Keywords:** inflammatory biomarkers, Interleukin-6, Interleukin-8, urine biosensor, non-faradaic electrochemical impedance spectroscopy

## Abstract

In this work, we propose a novel diagnostic biosensor that can enable stratification of disease states based on severity and hence allow for clear and actionable diagnoses. The scheme can potentially boost current Point-Of-Care (POC) biosensors for diseases that require time-critical stratification. Here, two key inflammatory biomarkers—Interleukin-8 and Interleukin-6—have been explored as proof of concept, and a four-class stratification of inflammatory disease severity is discussed. Our method is superior to traditional lab techniques as it is faster (<4 minutes turn-around time) and can work with any combination of disease biomarkers to categorize diseases by subtypes and severity. At its core, the biosensor relies on electrochemical impedance spectroscopy to transduce subtle inflammatory stimuli at the input for IL-8 and IL-6 for a limit of detection (LOD) of 1 pg/mL each. The biosensing scheme utilizes a two-stage random forest machine learning model for 4-state output disease classification with a 98.437% accuracy. This scheme can potentially boost the diagnostic power of current electrochemical biosensors for better precision therapy and improved patient outcomes.

## 1. Introduction

**Figure 1:**
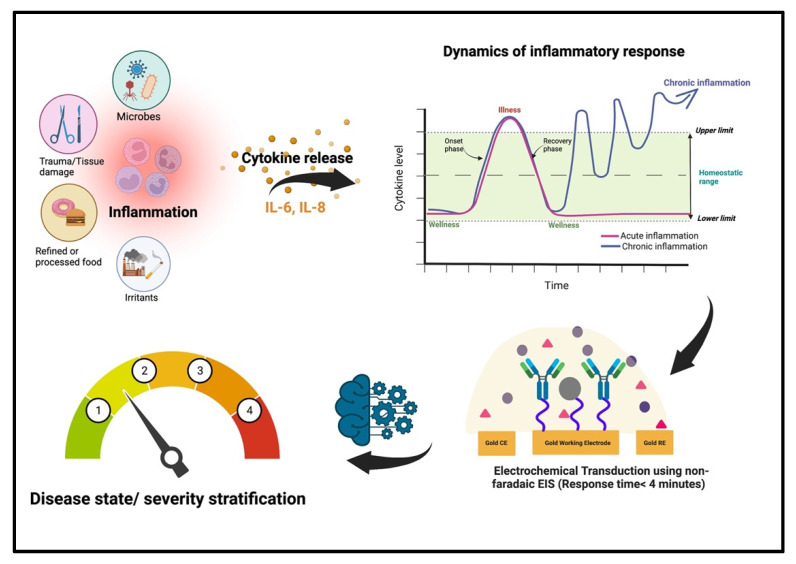
Schematic showing the operation of the proposed combinatorial inflammatory stimulus responsive biosensor. Created with BioRender.com (accessed on 4 September 2022).

In the precision medicine space, it has been realized that generalized treatment based on the phenotypic assessment of common symptoms, without considering the implications of underlying biological mechanisms, has been largely ineffective. Theragnostic are now being developed considering the heterogeneity underlying pathophysiological mechanisms among patients. This is especially important while treating a progressive inflammatory disorder or recurrent infectious disease, where it is imperative to consider the individuality of the immune system which can result in variability in disease onset and treatment response from one patient to another. In response to a harmful stimulus such as an infection or an injury, the body generates an inflammatory response and releases a cascade of chemical factors such as cytokines, chemokines, and other proteins to prevent further damage and restore function [1,2]. These biomolecules so released can either upregulate the inflammatory response further or can work towards down-regulating an exacerbated inflammatory process to restore balance or homeostasis (see Figure 1) [3]. By mapping the levels of these inflammatory mediators, the progress from wellness to illness due to disease onset and progression and back to wellness due to timely triage followed by appropriate therapy (for both acute and chronic inflammation) can be ensured [4].

For quantifying inflammation, urine has been widely studied in the research space, mainly because it is easily accessible, is “quiescent” since the molecular activity does not change much after sampling and does not require specialized sampling by trained professionals, and hence can be easily deployed for home-based routine screening [1,5]. There has been a great advancement in the field of POC diagnostic devices for inflammatory and infectious diseases in the current research space for a home-based multiplexed detection of biomarkers. These urine biosensors in their current formats output biomarker concentrations expressed in the urine samples without any information or interpretation of the severity of the disease and the urgency to schedule a doctor visit [6]. This defeats the very purpose of early detection through home-based diagnostics, as the patients are not trained medical professionals and hence cannot achieve a holistic understanding of their physiology when they are overwhelmed by looking at a bunch of numbers. Thus, it is important to come up with strategies to empower the patients by developing sensors that allow for comprehensive, reliable, and easy at-home stratification of inflammatory diseases.

To this end, we have developed a novel infectious/inflammatory-stimuli responsive combinatorial electrochemical biosensor for easy analysis and home-based diagnosis, stratification, prognosis, and therapy of diseases. The biosensor is versatile and can be extended to other inflammatory biomarkers associated with diseases that require time-critical stratification for efficacious treatment. To demonstrate proof of concept, we have chosen two established inflammatory biomarkers, viz., Interleukin-6 (IL-6) and Interleukin-8 (IL-8).

Interleukin-6 (IL-6) is a cytokine involved in the inflammatory response within the body and serves as an important biomarker for the early detection of various disease conditions such as systemic lupus erythematosus (SLE), wherein higher urinary IL-6 levels correspond to the disease behavior with the kidneys being affected by lupus nephritis [7]. Further, IL-6 levels in urine can also be used as an early indicator for Acute Kidney Injury (AKI) due to the damage of the proximal tubule characteristic of the disease [8]. IL-6 is produced mainly by monocytes, fibroblasts, endothelial cells, T cells, B cells, and mesangial cells [3]. Specifically, in lupus nephritis, macrophages and monocytes invade the cells in the kidney and produce IL-6. Since IL-6 is involved in the differentiation of B cells into antibody-producing plasma cells, it leads to the progression of the disorder as the antibodies lead to the attack of the host’s kidney cells. IL-6 also leads to the proliferation of mesangial cells, characteristic of LN, which secrete more IL-6 than seen through elevated levels in urine [9].

Interleukin-8 (IL-8) is another cytokine that serves as a versatile urinary biomarker for the detection of various diseases. IL-8 is secreted by fibroblasts, endothelial cells, monocytes, macrophages, and dendritic cells [10]. Urinary IL-8 is an important biomarker for the detection of acute pyelonephritis in febrile children, for prognosis of urinary bladder cancer, for non-Hodgkin’s lymphoma, and for detection of lupus nephritis [10,11]. Diagnosis of acute pyelonephritis is difficult in the pediatric population due to how the disease presents itself. This can lead to severe consequences of renal insufficiency and scarring if left undiagnosed [11]. Rapid and early detection of urinary IL-8 is critical to prevent these severe conditions. In urinary bladder cancer, IL-8 can be used to differentiate between superficial bladder cancer and muscle-invasive bladder cancer, where the more invasive the cancer is, the higher the levels of IL-8. Hence, a prognosis of this disease can be made in patients based on the measurement of IL-8 levels [11]. Urinary IL-8 levels are also important in the context of liver transplantation, where Acute Kidney Injury (AKI) is a frequent complication that can in some cases even lead to death. In a study, elevated post-operative levels of IL-8 were consistent with the presence of the disease [12].

The novelty of our sensor platform is that it stratifies the disease and outputs the disease stage associated with the patient sample at the input. This approach can be a paradigm shift in home-based POCs as it generates a single outcome from the quantitative analysis of multiple biomarkers (and not from a single biomarker-based semi-quantitative approach) but at the same time does not bog down the end user with the details of individual biomarkers levels. This makes the sensor output highly actionable and easy to read and interpret. This can help patients schedule doctor visits early on with greater confidence due to reduced chances of false negatives. This in turn can help the doctors in administering timely and accurate therapy which results in reduced costs, improved patient outcomes, and reduced hospital stay.

The developed sensor is based on affinity-based electrochemical biosensing wherein highly specific monoclonal antibodies have been used to capture the target antigens (IL-6 and IL-8) expressed in neat (unprocessed and unfiltered) urine samples. The sensor requires low sample volumes (~30 μL) and generates an output in less than 4 minutes. The transduction of the antibody-antigen binding event is achieved by monitoring the interfacial modulation of the electrode-urine interface as a function of the binding. The sensor is in the form of a standard planar gold three-electrode electrochemical system on a flexible, nanoporous lateral flow membrane substrate [5,13]. The porosity of the substrate allows for a wide dynamic range and enhanced sensitivity due to the phenomena and macromolecular crowding which is especially important for developing ultrasensitive sensors for detecting cytokines that are expressed in very low concentrations in biofluids [4,14,15]. The sensitivity of the sensor was further boosted by leveraging the powerful technique of electrochemical impedance spectroscopy (EIS) for electrochemical transduction of the binding events. The sensor was calibrated using EIS for IL-6 and IL-8 for a physiologically relevant wide dynamic range. Next, the optimized EIS parameters used for sensor calibration were utilized for disease state endotyping. It was demonstrated that our sensor is capable of handling multiple biomarkers and disease endotyping based on their corresponding levels expressed in urine. Next, the proposed concept was extended and fed to an established supervised machine learning model (2-stage random forest) for disease-based output classification. This is important when dealing with non-linear dependencies of complex samples and complicated biological pathways associated with dynamic inflammatory cascade events [16,17].

## 2. Materials and Methods

### 2.1. Materials and Reagents

The IL-6 and IL-8 monoclonal antibodies and antigen molecules were obtained from Abcam (Waltham, MA, USA). The Phosphate Buffer Saline (PBS) and the crosslinker DSP (dithiobis (succinimidyl propionate)) were obtained from Thermofisher Scientific Inc. (Waltham, MA, USA). Before the experiment, pooled human urine (PHU) samples ordered from Lee Biosolutions (St. Louis, MO, USA) are stored at −20 °C after being aliquoted. At the time of the experiment, the samples are thawed to room temperature and centrifuged to yield the supernatant solution used on the biosensor. Fusion 5 membrane from Cytiva (Global Life Sciences Solutions USA LLC, Marlborough, MA, USA) was used to create the lateral flow substrate for the electrochemical biosensor.

### 2.2. Sensor Fabrication and Electrode Modification

The biosensor was fabricated by a gold deposition process that involved a cryo-beam evaporator with a shadow mask on the Fusion 5 Cytiva lateral flow membrane. A lateral flow membrane was chosen for the sensor because of its ease of use, low cost, and efficiency of sensor fabrication. Of the lateral flow membranes, Fusion 5 was selected due to its fast-wicking rates, low background noise, a lack of need for blocking, and a lack of need for extra lateral flow assay components (e.g., blocking pad or absorbent pad) for the target analyte detection [13,18,19]. A standard, affinity-based, planar three-electrode electrochemical system composed of gold microelectrodes was used as the design for the biosensor. The thin layer of gold was deposited at a rate of 1 Å/s to achieve a thickness of 1500 Å. A backing made of plastic adhesive was used to structurally support the sensor. The electrode of the sensor was also modified using a primary amine-reactive DSP crosslinker that was used to bind the specific IL-6 and IL-8 antibodies to the gold electrodes via strong thiol bonds.

### 2.3. Design of EIS Studies

A Gamry Reference 6000 potentiostat (Gamry Instruments, Warminster, PA, USA) was used to conduct the electrochemical impedance spectroscopy experiments [20,21]. Data were collected across a large frequency range spanning 1 Hz–1 MHz while a voltage of 10 mVrms was applied to the working electrode. After each successive dose with a higher concentration was added to the sensor, the impedance value at 100 Hz (the value where the highest non-faradaic capacitive behavior was observed) was collected and compared to the impedance value for the zero dose (or the dose with no additional analyte concentration). The ratio of the impedance is then used to calibrate the sensor. The sensor calibration was performed for 1–100 pg/mL for IL-6 and 1–1000 pg/mL for IL-8 in human urine using EIS studies.

### 2.4. COMSOL Multiphysics Software Simulations

**Figure 2:**
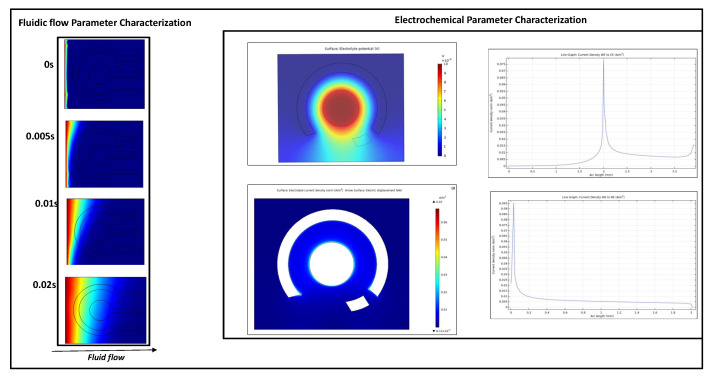
COMSOL study results for fluid flow and electrochemical parameter characterization.

COMSOL Multiphysics (COMSOL Multiphysics^®^ v5.4) software was used for finite element analysis to evaluate the fluid field and electric field characteristics of the electrochemical lateral flow biosensor (see Figure 2). The electric field simulation was conducted using the primary current distribution module to visualize both the electrolyte potential and the current distributions within the electrolyte.

The geometric models for analysis were designed using the AutoDesk™ AutoCAD software. The electrical model parameters were optimized and assigned based on the model described in our previous work [22]. Von Neumann boundary conditions were used to develop the geometric model. Fluid field analysis and electrode wicking simulations were carried out based on our previous work [23]. Transport of diluted species in porous media was used to develop the model.

### 2.5. Combinatorial Detection of IL-8 and IL-6: The Need for Urine Biomarker Benchmarking

To simulate the samples for different disease states, 4 different cocktails of the urine samples were prepared by 1:1 mixing samples with varying levels of both the biomarkers (i.e., IL-6 and IL-8) between 2 states, i.e., high (h) or low (L). Mathematically, for 2 biomarkers and 2 states of levels/inflammation, 2^2^ = 4 combinations are possible. These four states correspond to the output logical states for disease stratification and severity analysis.

### 2.6. Machine Learning-Based Disease Stratification Using Random Forest Model

The machine learning model was built on Google’s CoLab platform which uses a quad-core Intel Xeon processor running at 2.00 GHz per core, 24 gigabytes of ram, and a Tesla V100-SXM2-16GB GPU. The model has two stages of random forest classifiers. The first stage selects the most important features which are then used by the second stage RF model to classify the disease states. Sklearn library in Python was used, and bootstrapping was performed randomly with replacement. The optimal number of trees for the simple random forest model, i.e., RF1, and that for the optimized random forest, i.e., RF2 were chosen based on the number of trees that yield the maximum accuracy. The predictions of decision trees were bagged to get the final output class. The choice of random forest is dictated by the fact that this method is a power ensemble method (solves for the bias and high variance issues encountered in decision trees), works very well even with missing data, and is not affected drastically when new data are added. The capability to delineate the most important features based on random forest convergence was yet another important reason to choose it as the base learner model.

### 2.7. Statistical Analyses

Data are represented as mean ± SEM for *n* = 3 inter-sensor and *n* = 3 intra-sensor replicates. The significance test was carried out using Student’s *t*-test and 1-way ANOVA with α = 0.05. Non-linear regression and other statistical analyses were performed using Graph Pad Prism version 9.4.1 (GraphPad Software Inc., La Jolla, CA, USA). 

## 3. Results and Discussion

### 3.1. Design of the Combinatorial Biosensor

This work discusses the development of a simple, planar electrochemical system that does not require any microfluidic channels for fluid storage for reliable sensing. A standard three-electrode electrochemical system serves as the base electrode, while the urine buffer acts as the dielectric medium. The biosensor operates by transducing subtle variations in the impedance at the electrical double layer (using electrochemical impedance spectroscopy) due to the binding between the target cytokine (expressed picomolar concentration in urine) and its corresponding monoclonal antibody capture probe. Thus, it is important to model and optimize the electrical field behavior of the electrode system before performing the actual biosensing experiments to ensure suitable sensor calibration and to avoid denaturation of the biomolecules due to improper application of voltage (or electrical field). Since the sensor uses a combination of electrochemical analysis and lateral flow operation, the fluid field behavior was also studied to assess the operating conditions which were important as it reduced the iterative steps during assay development.

The sensor works with very low volumes of urine ~30 μL. Finite element analysis was used to simulate the fluid wicking in ideal conditions to optimize fluid volume and incubation times (porosity = 0.8). From Figure 2, we can observe rapid wicking in less than 0.05 s due to a capillary-driven lateral flow. COMSOL analysis was also used to optimize the design and the dimensions of the electrodes and the flexible lateral flow substrate. From Figure 2, it is evident that the highest potential occurs around (Working Electrode) WE, which decreases as the electrolyte travels outward. There is a significant decrease in potential when the electrolyte travels to the (Counter Electrode) CE compared to when it travels to the reference. Potential is at its lowest in the area that is covered by the CE. From the potential contour graphs, the electrolyte is at the highest at WE, which can be seen on the line graphs are well. The electrolyte potential graphs also show that there is a larger decrease in potential when the electrolyte travels towards CE compared to when it travels to the (Reference Electrode) RE. Additional details of the developed models have been discussed in the Supplementary Materials.

To study the capacitive properties of the electrical double layer (EDL) developed at the electrode-urine buffer interface as a result of analyte-capture probe binding, electrochemical impedance spectroscopy was used. This method is widely used for capacitive analysis especially in the field of biosensing as it is AC-based, compatible with small signal analysis, and suitable when working with proteins to avoid denaturation and compromise of structural integrity [24,25]. The use of urine as the diagnostic biofluid media affords several advantages. Urine, being highly ionic, serves as a high conductivity medium suitable for developing capacitive systems, and can be detected without significant electrode modification or redox tagging.

### 3.2. Calibration of the Sensor in Pooled Human Urine

**Figure 3:**
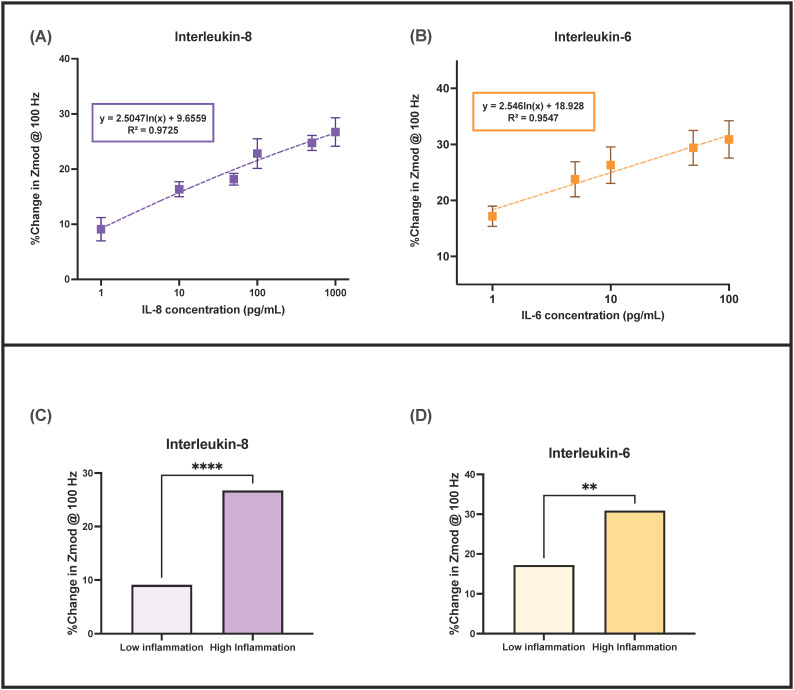
Calibration dose-response curve using EIS at 100 Hz for (**A**) IL-8 and (**B**) IL-6. Student’s *t*-test analysis between low inflammation and high inflammation for (**C**) IL-8 and (**D**) IL-6.

Modulation of dielectric properties at the electrode-urine buffer interface and a rearrangement of ions and water molecules, due to Ab-Ag binding were captured using a powerful AC-based electroanalytical technique, called electrochemical impedance spectroscopy (EIS). In this technique, a small sinusoidal AC voltage is applied at the working electrode and a phase-shifted current is obtained as the output. In EIS, the ratio of the input voltage to the output current gives a complex impedance characteristic of the biosensing system (correlated to the binding activity at a given analyte concentration). The physiologically relevant IL-8 level is 1–1000 pg/mL while that for IL-6 is 1–100 pg/mL in human urine. Figure 3 shows the dose-response curve for IL-6 and IL-8 detection in pooled human urine. A linear dose-response for synthetic urine (*n* = 3) and pooled human urine (*n* = 3), for a wide dynamic range, was observed for both IL-6 and IL-8 as shown in Figure 3A,B. The modulus of impedance decreases in a dose-dependent fashion as the system becomes increasingly capacitive due to binding. The Student’s *t*-test analysis results confirm that the sensor is capable of distinguishing between low and high levels of IL-6 and IL-8 in pooled human urine for inflammatory and non-inflammatory cases (*p*-value < 0.0001). In this way, the sensor was calibrated for urine IL-6 and IL-8 quantification.

### 3.3. Effect of Cross-Reactive Molecules and Urine pH on Electrochemical Sensor Performance

To evaluate the selectivity and specificity of the developed sensor, cross-reactivity experiments were performed. Cross-reactivity studies were conducted to study the impact of major constituents of urine, viz., urea and glucose on sensor performance. The student’s *t*-test analysis (two-tailed, unpaired) shows a <0.0001 for EIS meaning that there is a significant difference in the sensor response for the same concentration of the specific, i.e., IL-6 and IL-8) and non-specific urine constituents (i.e., glucose and urea). Thus, from these experiments, it is evident that the system is specific and selective for the target biomarkers. The signal for IL-6 and IL-8 does not cross-react with that due to interferent molecules and the sensor is able to significantly discriminate between inflammation and non-inflammation states. 

### 3.4. Precision Analysis

**Figure 4:**
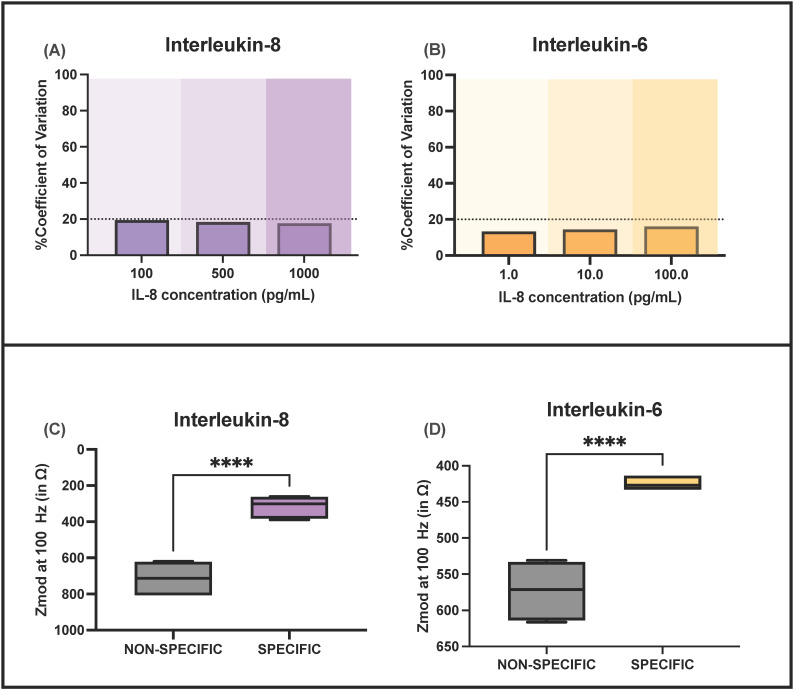
CV%-based precision analysis of the sensor for (**A**) IL-8 and (**B**) IL-6. Interference study analysis using urea and glucose for (**C**) IL-8 and (**D**) IL-6.

To evaluate the precision of EIS for both the biomarkers, the coefficient of variation (CV%) was calculated by averaging over all the replicates as a function of the IL-6 and IL-8 levels spiked in urine samples for low, medium, and elevated biomarker levels (see Figure 4). It was found that the CV% of the doses falls well within the acceptable range of 20% set by Clinical Laboratory Standards Institute (CLSI) guidelines [26,27]. In this way, it was concluded that the sensor is capable of precise measurements of urine samples for both biomarkers.

### 3.5. Implementation of the Sensor Using Supervised Machine Learning Platform

**Figure 5:**
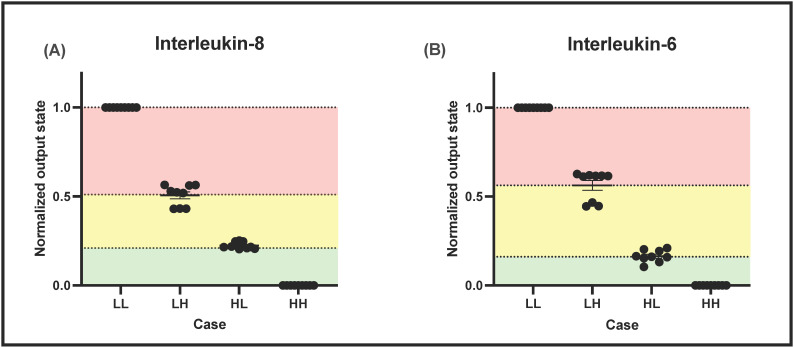
Normalization of the sensor output impedance into disease states for (**A**) IL-8 and (**B**) IL-6.

Cocktail solutions corresponding to 4 output states were tested using EIS at 100 Hz on both the developed IL-8 and IL-6 sensors. The four output states correspond to “healthy”, “inflammation, pre-symptomatic”, “inflammation, symptomatic”, and “inflammation, systemic” states, respectively. The 4 states were designated as LL, LH, HL, and HH where the first letter corresponds to IL-8 and the second letter corresponds to IL-6 levels; L means low and H means high. For example, for state 2, i.e., state “LH”, the level of IL-8 is low and the level of IL-6 is high. For IL-6, “L” or low corresponds to 1 pg/mL and “H” or high corresponds to 100 pg/mL. For IL-8, “L” or low corresponds to 1 pg/mL and “H” or high corresponds to 5000 pg/mL. From Figure 5A,B, upon normalization, it was observed that the sensor was able to clearly delineate between the stages of inflammation for both IL-6 and IL-8 sensors. In this way, the progress of inflammation, from LL (output state = 1), LH (output state = 1), HL (output state = 1), and HH (output state = 1) can reliably be tracked and programmed into the biosensor. This model can be further extended to more biomarker combinations, depending on the disease model and stratification required.

To further boost the stratification, the impedance data were then trained and tested using a simple machine learning model. To avoid high computation costs, the random forest (RF) method was developed which uses multiple decision trees with varying numbers and depths to achieve mapping of relationships between the inputs and the outputs. RF models are often used in real-world research problems as they are highly immune to the effects of biases and variances because they give out a classification based on bootstrapping the outputs of multiple decision trees, instead of relying on a single tree [28].

The sensor data acquired from the two biomarkers corresponding to the four stratification states were labeled as ‘1’, ‘2’, ‘3’, or ‘4’ corresponding to the output digital state. The real, imaginary, modulus, and phase values of impedance obtained for each target analyte were studied along at 100 Hz for both IL-6 and IL-8. 

**Figure 6:**
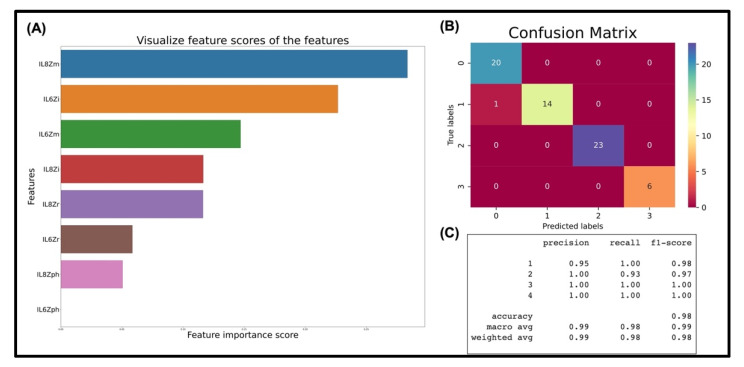
Machine learning analysis. (**A**) shows the score or ranking of the input features, (**B**) confusion matrix, and (**C**) other metrics of the developed machine learning model for disease state stratification.

The total size of the dataset was 318 rows and 9 columns. The RF model was tuned to obtain the maximum number of trees to achieve maximum accuracy for the given dataset. It was found that for the given dataset, 2 trees obtained the highest accuracy of 98.4375% using 80% of the dataset for training and 20% of the dataset for testing. The confusion matrix in Figure 6B shows the output from a single random forest. Most of the results appear in the main diagonal, meaning that the model was capable of correctly predicting and classifying the output disease states with high accuracy.

Next, the model was used to analyze the importance of the different features for UTI endotyping. Figure 6A shows the bar graphs of the features in descending order of importance. After identification of the key features (discussed in detail in the supplementary information), a new random forest model (random forest 2) was developed wherein only the 3 most important features were studied and the relatively less important feature was dropped. For the reduced and optimized random forest, i.e., RF-2, fewer trees, i.e., a total of 2 trees were required to reach the same maximum accuracy of ~98%, keeping the tree depth constant. To study the metrics corresponding to the ability of the model to classify the four digital output states, ‘precision’, ‘recall’, and F-1 scores were studied. The values of the metrics have been listed in Figure 6C for all four classes. In this way, the implementation of target disease stratification and endotyping was demonstrated using a simple statistical machine learning algorithm. To our knowledge, this is the first demonstration of direct integration of raw impedance values from an electrochemical biosensor to train a machine learning model for multistate disease classification. As mentioned before, this model is versatile and can be extended to more complicated disease states with more stratification levels by studying a broader panel of biomarkers.

## 4. Conclusions

This work is the first technological demonstration of a lateral flow-based electrochemical biosensor for combinatorial quantification of inflammatory biomarkers in urine for disease severity stratification. This work uses EIS to evaluate the sensitivity of the platform for a wide dynamic range of −100 pg/mL for IL-6 and 1–1000 pg/mL for IL-8 for very low volumes (<30 μL) of urine within 4 minutes. Our sensor is capable of handling multiple biomarkers and disease endotyping based on their corresponding levels expressed in urine. The biosensing concept is versatile and modular and can be applied to any disease model which requires time-critical stratification for efficacious treatment. Notwithstanding that there are several biomarkers associated with inflammation, we have focused on two established inflammatory urinary biomarkers for our proof-of-concept study viz., IL-6 and IL-8. The novelty of our sensor platform is that it stratifies illness progression and gives out a digital state output corresponding to disease endotype. This is a first-of-a-kind biosensor that endotypes and classifies disease states based on severity considering levels of host inflammatory biomarkers. Non-faradaic, label-free EIS was used to study the subtle interfacial modulation due to the specific affinity capture of the target analytes IL-6 and IL-8 by their corresponding highly specific monoclonal antibody. Four disease states were studied and arbitrarily assigned outcomes, viz., states 1 through 4 corresponding to “healthy”, “inflammation, pre-symptomatic”, “inflammation, symptomatic”, and “inflammation, systemic.” These states were chosen arbitrarily, and the proposed model can be extended to more biomarkers and varied classification states. The sensor can be extended for endotyping other complex and heterogenous diseases in other body fluids and tissue samples. This sensor demonstrates the use of the random forest as the algorithm for machine-guided classification of the output disease states. However, given the nature of the signal extracted from the electrochemical sensor, and the complexity of the events at the double layer interface, the dataset can be further augmented, and other traditional and deep learning statistical models can be employed considering the tradeoff between application and the computational costs post-implementation as POC device.

## Data Availability

The data presented in this study are available on request from the corresponding author.

## Supplementary Materials

The following supporting information can be downloaded at: https://www.mdpi.com/article/10.3390/s22207757/s1, Table S1. Features studied using Random-forest model; Figure S1: Bode Magnitude and Bode Phase plots showing impedance response for Interleukin-6 and Interleukin-8 with dosing; Figure S2: Receiver Operating Characteristic curve for the developed Random-forest model; Figure S3: CAD design and image of the bare gold electrodes (placed next to a US quarter dollar coin for size comparison); Figure S4: Design of the COMSOL model; Figure S5: Cut line from WE to CE; Figure S6: Cut line from WE to RE; Figure S7: Line graph for electrolyte for WE to CE; Figure S8: Line graph for electrolyte for WE to RE; Figure S9: Primary current distribution equations (Electrochemistry module); Figure S10: Electric current equations-Stationary analysis (Electrochemistry module); Figure S11: Fluid flow equation-Time dependent study (Porous media flow module). References [29,30] are cited in the supplementary materials.

## References

[1] Prasad S., Tyagi A.K., Aggarwal B.B. (2016). Detection of Inflammatory Biomarkers in Saliva and Urine: Potential in Diagnosis, Prevention, and Treatment for Chronic Diseases. Exp. Biol. Med..

[2] Tosi M.F. (2005). Innate Immune Responses to Infection. J. Allergy Clin. Immunol..

[3] Turner M.D., Nedjai B., Hurst T., Pennington D.J. (2014). Cytokines and Chemokines: At the Crossroads of Cell Signalling and Inflammatory Disease. Biochim. Biophys. Acta-Mol. Cell Res..

[4] Jagannath B., Lin K.C., Pali M., Sankhala D., Muthukumar S., Prasad S. (2021). Temporal Profiling of Cytokines in Passively Expressed Sweat for Detection of Infection Using Wearable Device. Bioeng. Transl. Med..

[5] Ganguly A., Ebrahimzadeh T., Zimmern P.E., De Nisco N.J., Prasad S. (2021). Label Free, Lateral Flow Prostaglandin E2 Electrochemical Immunosensor for Urinary Tract Infection Diagnosis. Chemosensors.

[6] Ganguly A., Ebrahimzadeh T., Zimmern P., De Nisco N.J., Prasad S. (2022). Label-Free, Novel Electrofluidic Capacitor Biosensor for Prostaglandin E2 Detection toward Early and Rapid Urinary Tract Infection Diagnosis. ACS Sens..

[7] Petrovszki D., Valkai S., Gora E., Tanner M., Bányai A., Fürjes P., Dér A. (2021). An Integrated Electro-Optical Biosensor System for Rapid, Low-Cost Detection of Bacteria. Microelectron. Eng..

[8] Su H., Lei C.T., Zhang C. (2017). Interleukin-6 Signaling Pathway and Its Role in Kidney Disease: An Update. Front. Immunol..

[9] Dennen P., Altmann C., Kaufman J., Klein C.L., Andres-Hernando A., Ahuja N.H., Edelstein C.L., Cadnapaphornchai M.A., Keniston A., Faubel S. (2010). Urine Interleukin-6 Is an Early Biomarker of Acute Kidney Injury in Children Undergoing Cardiac Surgery. Crit. Care.

[10] Tackey E., Illei G.G. (2004). Rationale for Interleukin-6 Blockade in Systemic Lupus Erythematosus. Lupus.

[11] Aragón C.C., Tafúr R.A., Suárez-Avellaneda A., Martínez M.T., de las Salas A., Tobón G.J. (2020). Urinary Biomarkers in Lupus Nephritis. J. Transl. Autoimmun..

[12] Shahzad A., Knapp M., Lang I., Köhler G. (2010). Interleukin 8 (IL-8)-a Universal Biomarker?. Int. Arch. Med..

[13] Sirota J.C., Walcher A., Faubel S., Jani A., McFann K., Devarajan P., Davis C.L., Edelstein C.L. (2013). Urine IL-18, NGAL, IL-8 and Serum IL-8 Are Biomarkers of Acute Kidney Injury Following Liver Transplantation. BMC Nephrol..

[14] Whatman Fusion 5|Cytiva. https://www.cytivalifesciences.com/en/us/shop/whatman-laboratory-filtration/whatman-dx-components/lateral-flow-pads/fusion-5-p-00787#tech-spec-table.

[15] Liu C., Chu D., Kalantar-Zadeh K., George J., Young H.A., Liu G. (2021). Cytokines: From Clinical Significance to Quantification. Adv. Sci..

[16] Kamakoti V., Kinnamon D., Choi K.H., Jagannath B., Prasad S. (2018). Fully Electronic Urine Dipstick Probe for Combinatorial Detection of Inflammatory Biomarkers. Futur. Sci. OA.

[17] Cui F., Yue Y., Zhang Y., Zhang Z., Zhou H.S. (2020). Advancing Biosensors with Machine Learning. ACS Sens..

[18] Schackart K.E., Yoon J.Y. (2021). Machine Learning Enhances the Performance of Bioreceptor-Free Biosensors. Sensors.

[19] Parolo C., Sena-Torralba A., Bergua J.F., Calucho E., Fuentes-Chust C., Hu L., Rivas L., Álvarez-Diduk R., Nguyen E.P., Cinti S. (2020). Tutorial: Design and Fabrication of Nanoparticle-Based Lateral-Flow Immunoassays. Nat. Protoc..

[20] Electrochemical Impedance Spectroscopy Gamry Instruments. https://www.gamry.com/application-notes/EIS/.

[21] Ganguly A., Lin K.-C., Muthukumar S., Nagaraj V.J., Prasad S. (2021). Label-Free Protein Glycosylation Analysis Using NanoMonitor—An Ultrasensitive Electrochemical Biosensor. Curr. Protoc..

[22] Stevenson H., Radha Shanmugam N., Paneer Selvam A., Prasad S. (2018). The Anatomy of a Nonfaradaic Electrochemical Biosensor. SLAS Technol..

[23] Upasham S., Thai K., Muthyala R., Prasad S. (2020). Flexible, Low Volume Detection of Chronobiology Biomarkers from Human Sweat. Analyst.

[24] Randviir E.P., Banks C.E. (2013). Electrochemical Impedance Spectroscopy: An Overview of Bioanalytical Applications. Anal. Methods.

[25] Daniels J.S., Pourmand N. (2007). Label-Free Impedance Biosensors: Opportunities and Challenges. Electroanalysis.

[26] EP15A3 User Verification of Precision & Bias Estimation. https://clsi.org/standards/products/method-evaluation/documents/ep15/.

[27] CLSI EP05-A2—Evaluation of Precision Performance of Quantitative Measurement Methods; Approved Guideline—Second Edition. https://webstore.ansi.org/Standards/CLSI/clsiep05a2.

[28] Conev A., Litsa E.E., Perez M.R., Diba M., Mikos A.G., Kavraki L.E. (2020). Machine Learning-Guided Three-Dimensional Printing of Tissue Engineering Scaffolds. Tissue Eng. -Part A.

[29] (1998). Electrochemistry Module User’s Guide. https://doc.comsol.com/5.3/doc/com.comsol.help.echem/ElectrochemistryModuleUsersGuide.pdf.

[30] (1998). Porous Media Flow Module. https://doc.comsol.com/5.5/doc/com.comsol.help.porous/IntroductionToPorousMediaFlowModule.pdf.

