# Supplementary material for: Inflammatory Stimuli Responsive Non-Faradaic, Ultrasensitive Combinatorial Electrochemical Urine Biosensor
Source: Sensors (Basel). 2022 Oct 13;22(20):7757. doi: 10.3390/s22207757 (PMC9611881; doi:10.3390/s22207757)
Supplement: Supplementary file 1 [file sensors-22-07757-s001.zip › sensors-1926763-supplementary.pdf]

Inflammatory stimuli responsive Non-faradaic, Ultrasensitive combinatorial electrochemical Urine biosensor

Antra Ganguly [1], Varun Gunda [1], Kevin Thai [1] and Shalini Prasad [1,*]

1 Department of Bioengineering, The University of Texas at Dallas
* Correspondence: Shalini.Prasad@utdallas.edu

Table S1. Features studied using Random-forest model.

Feature #	Feature name	Description
1	IL8Zr	Zreal or real component of impedance for IL-8
2	IL8Zi	Zimag or imaginary component of impedance
3	IL8Zm	Zmod or modulus of impedance for IL-8
4	IL8Zph	Zphase or phase angle (in degrees) of impedance for IL-8
5	IL6Zr	Zreal or real component of impedance for IL-6
6	IL6Zi	Zimag or imaginary component of impedance for IL-6
7	IL6Zm	Zmod or modulus of impedance for IL-6
8	IL6Zph	Zphase or phase angle (in degrees) of impedance for IL-6

Variation in impedance response for Interleukin-6 and Interleukin-8 with dosing

To study the modulation of the electric double layer at the sensor interface, characteristic Bode magnitude and Bode phase response plots were analyzed. In Bode magnitude plot, the modulus of impedance (measured in Ohms) is plotted on the Y-axis, while logarithm of the frequency (measured in Hertz) is plotted on the X-axis. In Bode phase plot, the phase of impedance (measured in degrees) is plotted on the Y-axis, while logarithm of the frequency (measured in Hertz) is plotted on the X-axis.
In figure S2 shown below, a wide frequency range from 10-1000 Hz has been shown for the entire dynamic range for IL-6 (1-100 pg/mL) and IL-8 (1-1000 pg/mL) to characterize the sensor response. The Bode magnitude plots have been depicted as solid lines with symbols while the phase plots have been plotted as plain lines without symbols. From the Bode magnitude plots, a dose dependent change in

the impedance modulus is observed at a given frequency (including the frequency of operation i.e., 100 Hz) for both IL-6 and IL-8. Also, from the Bode phase plots, it can be clearly observed that the sensor shows negative phase (tends towards -90 degrees) for IL-6 and IL-8, it can be observed that the sensor operates in the capacitive region at 100 Hz.

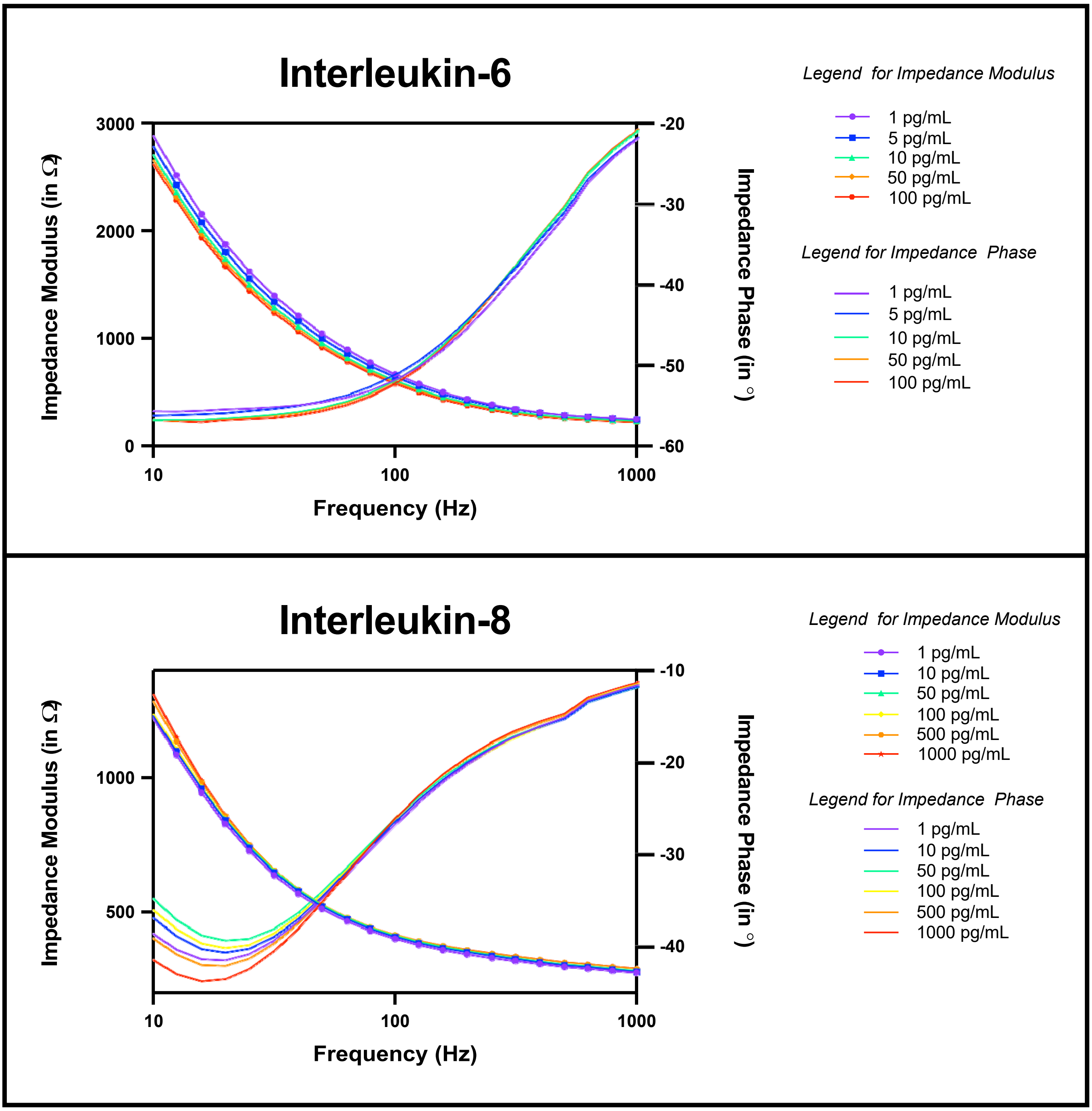

Figure S1: Bode Magnitude and Bode Phase plots showing impedance response for Interleukin-6 and Interleukin-8 with dosing

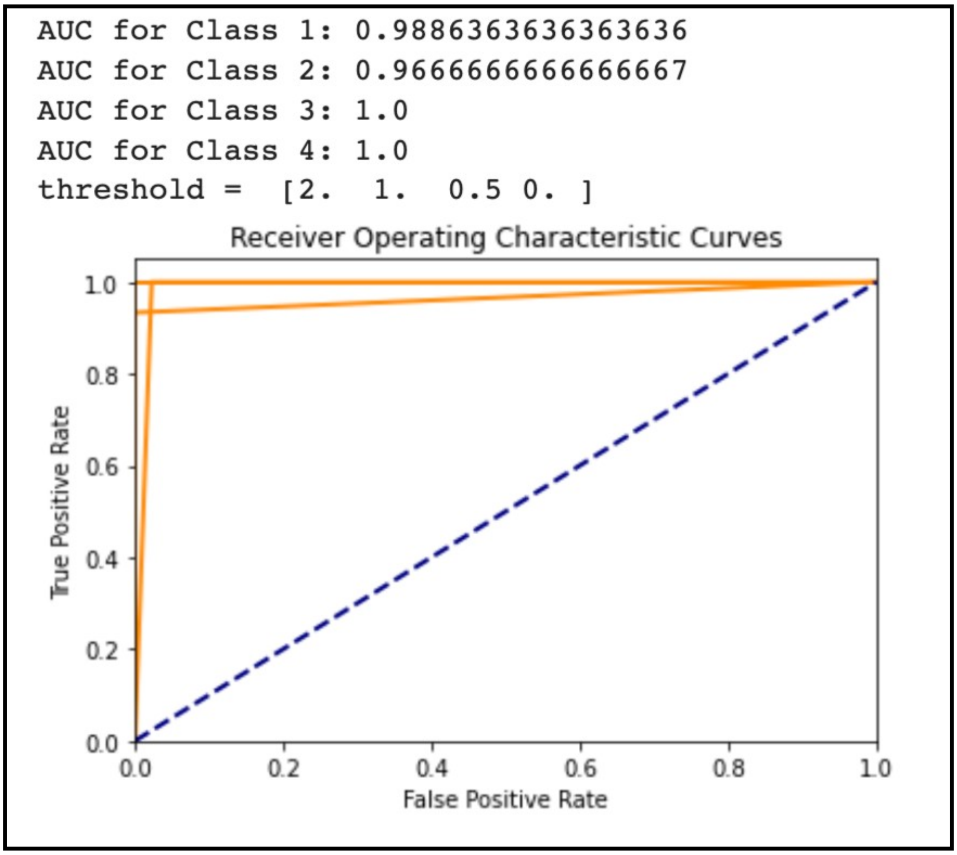

Figure S2: Receiver Operating Characteristic curve for the developed Random-forest model

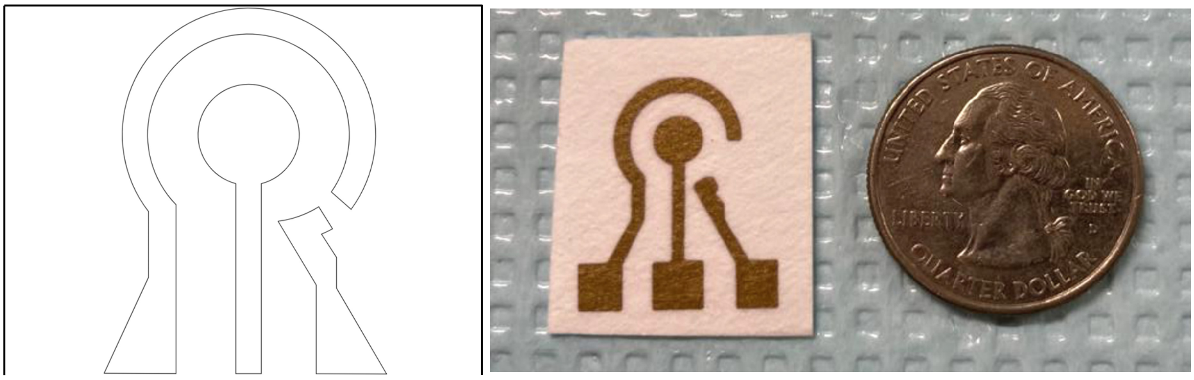

Figure S3: CAD design and image of the bare gold electrodes (placed next to a US quarter dollar coin for size comparison)

Details of the developed COMSOL Multiphysics models

COMSOL Multiphysics was utilized to perform Finite Element analysis (FEA) of the sensor electrode design. The model has two parts, a PBS block and an electrode that is placed completely inside the PBS block. The electrode has three parts, working electrode (WE), counter electrode (CE), and reference. The WE has a round geometry and its center can be seen as the origin. It has a diameter of 4 mm. The CE is a curve, with a width of 1.25 mm, that revolves approximately 225 degrees around the origin. The distance between the origin and CW is 2 mm. The reference is built similar to CE and it revolves

approximately 30 degrees around the origin. The gap between CW and reference is no longer than 0.75 mm. Every part of the electrode has a thickness of 0.015 mm. The PBS block has a thickness of 0.5 mm, width of 14 mm, and height of 12 mm.

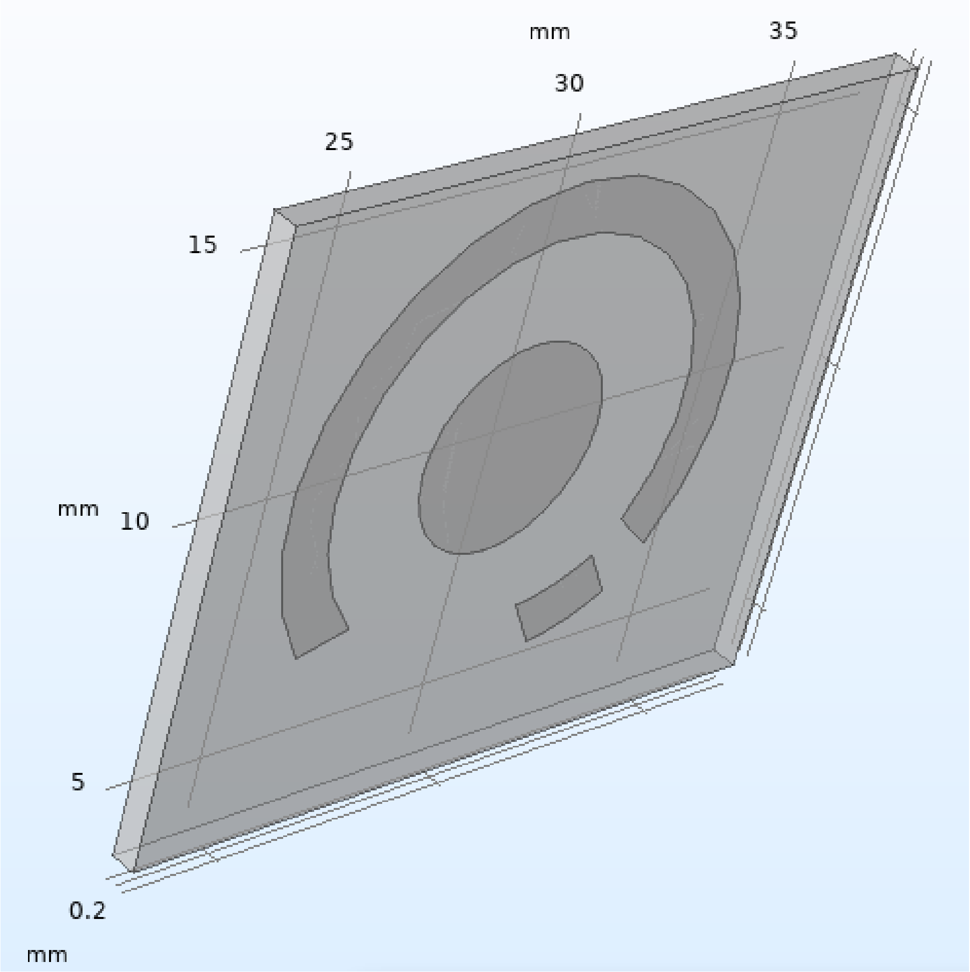

Figure S4: Design of the COMSOL model

The electrode system is at the center and is completely submerged into a PBS block. The whole model is set under room temperature, which is close to 293.15 K. The electrode is made up of gold, which has a relative permittivity of 4.1E7. The block of PBS has a relative permittivity of 80. An electrode potential of 0.01 V is applied on the working electrode (WE). The reference impedance is set to 50 Ohm. Electrolyte potential is measured for the electrode using separate intervals and with it, the current density is approximated. Figure S6 and S7, shows the cut lines from WE to CE and WE to RE and figure S8 and S9 show the corresponding line graphs for the electrolyte potential.

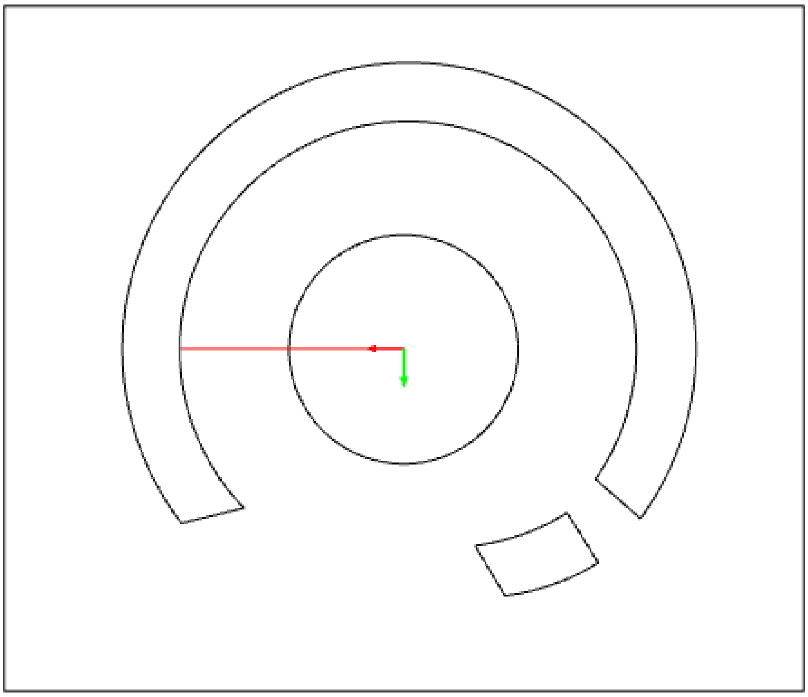

Figure S5: Cut line from WE to CE

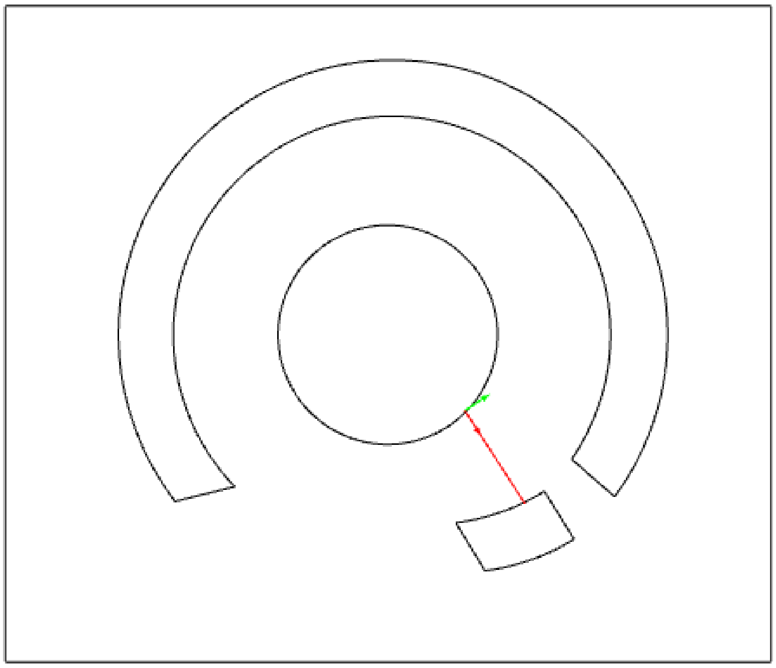

Figure S6: Cut line from WE to RE

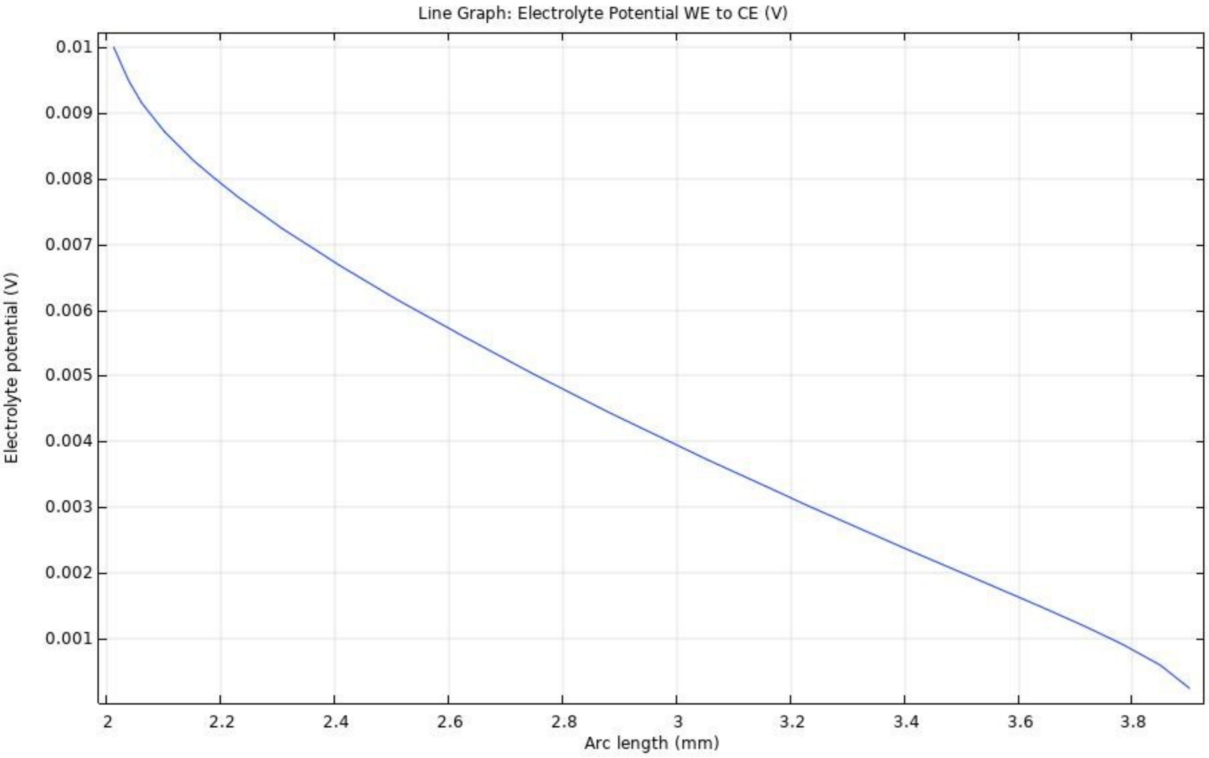

Figure S7: Line graph for electrolyte for WE to CE

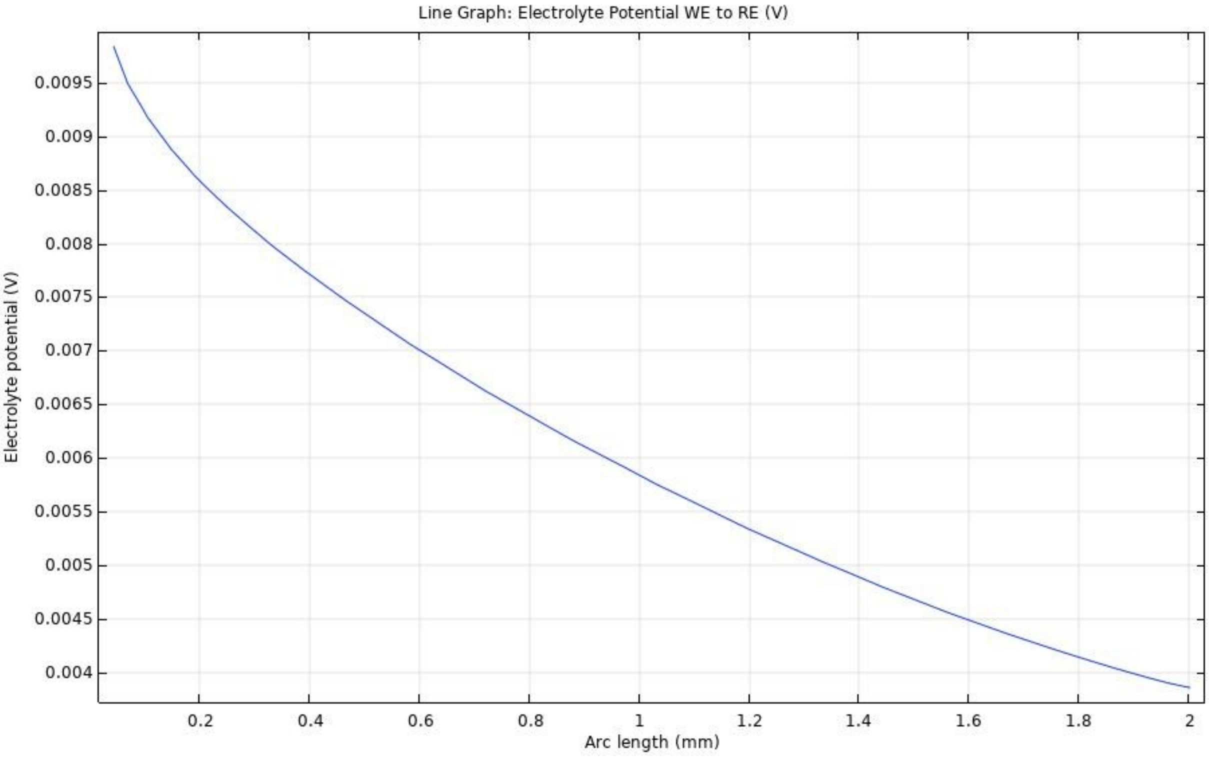

Figure S8: Line graph for electrolyte for WE to CE

The transport of dilute species in porous media physics in COMSOL Multiphysics® was utilized to analyze the concentration distribution as a function of time, where the lateral flow membrane (Cytiva Fusion 5) was represented as the porous medium. The results of the simulations have been shown in figure 2 of the main manuscript. The underlying assumptions are (i) the porous medium is homogeneous without internal frictional loss and is (ii) at atmospheric pressure and room temperature. The simulation results shown in Fig. 2 (main manuscript) depicts the simulated concentration distribution through the porous media at 0 s to 0.02 s to illustrate the homogenous and rapid wicking profile. It was observed if the fluid (urine containing the target biomarkers i.e., IL-6 or IL-8) is introduced at the sensor at time t=0s, it takes less than 1 s (nearly 0.02 s) for it to reach the working electrode and interact with its corresponding antibody capture probe. This is desirable to ensure a fast turnaround time for sensor readout for rapid sensing.

The equations corresponding to primary current distribution, electrical current analysis and fluid flow analysis have been depicted in figure S10-S12 respectively. The notations and variable names have their default meanings[1,2].

$$\nabla \cdot \mathbf{i}_l = Q_l, \quad \mathbf{i}_l = -\sigma_l \nabla \phi_l$$
$$\nabla \cdot \mathbf{i}_s = Q_s, \quad \mathbf{i}_s = -\sigma_s \nabla \phi_s$$
$$\phi_l = \text{phil}, \quad \phi_s = \text{phis}$$

Figure S9: Primary current distribution equations (Electrochemistry module)

$$\nabla \cdot \mathbf{J} = Q_{j,v}$$
$$\mathbf{J} = \sigma \mathbf{E} + \mathbf{J}_e$$
$$\mathbf{E} = -\nabla V$$

Figure S10: Electric current equations-Stationary analysis (Electrochemistry module)

$$\frac{\partial(\epsilon_p c_i)}{\partial t} + \frac{\partial(\rho c_{P,i})}{\partial t} + \nabla \cdot \mathbf{J}_i + \mathbf{u} \cdot \nabla c_i = R_i + S_i$$
$$\mathbf{J}_i = -D_{e,i} \nabla c_i$$
$$\theta = \epsilon_p$$

Figure S11: Fluid flow equation-Time dependent study (Porous media flow module)

References:

1. Electrochemistry Module User's Guide. **1998**.
2. Porous Media Flow Module. **1998**.
